# Characterization of Khorasan wheat (Kamut) and impact of a replacement diet on cardiovascular risk factors: cross-over dietary intervention study

**DOI:** 10.1038/ejcn.2012.206

**Published:** 2013-01-09

**Authors:** F Sofi, A Whittaker, F Cesari, A M Gori, C Fiorillo, M Becatti, I Marotti, G Dinelli, A Casini, R Abbate, G F Gensini, S Benedettelli

**Affiliations:** 1 Agency of Nutrition, Careggi University Hospital, Florence, Italy; 2 Department of Medical and Surgical Critical Care, Thrombosis Centre, University of Florence, Florence, Italy; 3 Don Carlo Gnocchi Foundation Florence, Florence, Italy; 4 Department of Agronomy and Land Management, University of Florence, Florence, Italy; 5 Department of Biochemical Sciences, University of Florence, Florence, Italy; 6 Department of Agroenvironmental Science and Technology, University of Bologna, Bologna, Italy; 7 Interdipartimental Center for Research on Food and Nutrition, University of Florence, Florence, Italy

**Keywords:** grain, Kamut, cardiovascular disease, risk factors, diet

## Abstract

**Background/Objectives:**

Khorasan wheat (Kamut) is an ancient grain with widely acclaimed beneficial effects on human health. The objective was to characterise Kamut and to examine the effect of a replacement diet with their products on cardiovascular risk parameters.

**Subjects/Methods:**

We conducted a randomized, single-blinded cross-over trial with two intervention phases on 22 healthy subjects (14 females; 8 males). The participants were assigned to consume products (bread, pasta and crackers) made either from Kamut or control semi-whole-grain wheat for 8 weeks in a random order. An 8-week washout period was implemented between the interventions. Laboratory analyses were performed both at the beginning and at the end of each intervention phase.

**Results:**

At a general linear model for repeated measurements adjusted for several confounders, consumption of Kamut products showed a significant reduction of metabolic risk factors such as total cholesterol (mean reduction: −8.46 mg/dl; −4%), low-density lipoprotein cholesterol (−9.82 mg/dl; −7.8%) and blood glucose. Similarly, redox status was significantly improved only after the Kamut intervention phase, as measured by a reduction in both thiobarbituric acid reactive substances (−0.17 nmol/ml; −21.5%) and carbonyl levels (−0.16 nmol/ml; −17.6%). The replacement diet with Kamut products also resulted in a significant increase of serum potassium and magnesium. Circulating levels of key pro-inflammatory cytokines (interleukin (IL)-6, IL-12, tumour necrosis factor-α and vascular endothelial growth factor) were significantly reduced after the consumption of Kamut products.

**Conclusions:**

The present results suggest that a replacement diet with Kamut products could be effective in reducing metabolic risk factors, markers of both oxidative stress and inflammatory status.

## Introduction

Wheat is considered one of the most important components of human nutrition, and on a global scale provides a source of dietary carbohydrates, proteins, vitamins, minerals and fibre.^1, 2, 3, 4^ Whole-grain consumption, in particular, has been reported to have a protective role against cardiovascular disease, diabetes and cancer.^3, 4, 5, 6^ Recently, research interest has been focused on ancient wheat grain varieties, previously recognised as a rich source of health-promoting substances.^7^ Among the ancient grain varieties, Khorasan wheat (Kamut) has emerged as one of the most important for distribution and marketing.

Kamut has recently been found to have the potential to promote the growth of probiotic strains in the gastrointestinal tract.^8^ Moreover, it has been also shown that bread made from Kamut protects rats from oxidative stress to a greater extent than that afforded by whole-grain durum wheat.^9^ Despite widespread publicity regarding the potential health benefits of Kamut, to the best of our knowledge no study has been conducted to evaluate the health-promoting effects of Kamut products on humans. The aim of this intervention study was to characterise Kamut and to test the efficacy of a replacement diet with Kamut products on risk parameters of cardiovascular and metabolic diseases.

## Materials and methods

### Study population

In all, 22 healthy volunteers (8 men, 14 women; mean±s.d. age: 50.5±11.8 years) with a body mass index (BMI; in kg/m^2^) between 17.1 and 31.6 (mean±s.d. BMI: 23.3±3.6) were recruited from the staff of the University of Florence and from their family/friends.

Inclusion criteria for subject participation in the study necessitated that participants had to: be of an age ranging between 20 and 70 years, be in good general health, be neither pregnant nor lactating and have neither a gluten allergy nor gastrointestinal disorders (e.g. chronic constipation, diarrhoea, inflammatory bowel disease, irritable bowel syndrome or other chronic gastrointestinal complaints) and gall bladder problems. Volunteers were instructed not to alter their usual dietary or fluid intakes.

### Wheat varieties

Organically grown Kamut brand Khorasan wheat, obtained from Sasketechewen (Canada), the principle cultivation area, was utilised as the experimental wheat under investigation. The seed was milled by Molino SIMA S.C.A.R.L. (Argenta, Ferrara, Italy) and both semi-whole-wheat semolina and flour were purchased. As the control, semi-whole-wheat semolina and wheat flour, respectively, derived from a mix of modern Italian durum and soft wheat varieties were purchased from the same mill. As with the Kamut, both the Italian soft and durum wheat varieties, from hereon referred to as the control, had similarly been cultivated under organic agricultural conditions. To further standardise the comparison, all transformation preparation procedures were identical for both the Kamut (experimental) and control wheat under study. Pasta was prepared from the Kamut and durum wheat by the Pastificio Artigiano FABBRI s.a.s. (Strada in Chianti, Firenze, Italy), an artisan pasta maker, whereas the bread, biscuits and crackers were made from the Kamut and soft wheat by the artisan enterprise of Panificio Menchetti Pietro di Santi e Figli s.n.c. (Cesa Marciano della Chiana, Arezzo, Italy).

### Study design

The study was a randomized, single-blinded, cross-over trial designed to test whether a replacement diet with grain products made from Kamut would benefit the cardiovascular risk profile of the participants, compared with a similar replacement diet using grain products made from organic, semi-whole-grain wheat durum and soft wheat varieties. After a 2-week run-in period, the eligible participants were randomly divided into two groups (11 individuals/group), each assigned to consume either the Kamut or control products, respectively. Starting from June 2011, participants in both groups then received 500 g/week of pasta, 150 g/day of bread, 500 g/month of crackers and 1 kg/month of biscuits for a period of 8 weeks. In the intervention period, all participants were not permitted to eat other grains products. A washout period of 8 weeks was then implemented, in which participants were permitted to eat all foods according to their ‘normal eating habits'. From October 2011, the second intervention phase was implemented with the group, assigned to consume the control products in the first phase, now assigned to consume the Kamut products, and *vice versa*. At baseline and after each intervention, all subjects were examined between 0700 hours and 0930 hours after a 12-h-fasting period. Furthermore, subjects were asked not to engage in strenuous physical activity during the day before the examination. BMI was calculated as weight (kg)/height (m)^2^. Written informed consent was obtained from each participant before the initial screening visit and before randomisation. The institutional review board at the University of Florence approved the study protocol.

### Characteristics of the wheat varieties

Insoluble dietary fibre and soluble dietary fibre were measured according to the instruction protocol provided by the Megazyme Total Dietary Fibre Assay Procedure Kit (Megazyme International, Co. Wicklow, Ireland), based on the methods of Prosky *et al.*^10^ and Lee *et al.*^11^ The protein content, associated with the insoluble and soluble cell wall polysaccharide fractions, respectively (and which was subtracted from the gravimetric mass of both dried insoluble dietary fibre and ethanol-precipitated dried soluble dietary fibre fractions), was calculated from the N content. N content (nearly 2.0 mg) was measured in the dried samples using the Flash Elemental Analyser 1112 NC (Thermo Fisher Scientific, Waltham, MA, USA) according to the manufacturer's instructions. Both resistant starch and non-resistant starch were determined according to the enzymatic procedure outlined by the instruction manual for the Megazyme-Resistant Starch Assay Procedure Kit (Megazyme International, Co.), based on the method of McClerry and Monaghan.^12^ The amylase and amylopectin contents were similarly determined using the Megazyme Amylose/Amylopectin Assay Kit (Megazyme International, Co) according to a modification of the method developed by Yun and Matheson.^13^ The macro- (Ca, K, Mg and P) and micro-elements (Fe, Se, V and Zn) were extracted by nitric acid digestion (0.5 g in 10 ml 7:3 (v/v) nitric acid: H_2_O) in a Microwave Accelerated Reaction System (Model MARS, CEM Cooperation, North Carolina, USA). Digestion conditions consisted of three ramps: 120 °C (1 min), 170 °C (1 min) and 180 °C (10 min) in which 12, 6 and 4 min were required to arrive at the respective maximum efficiency temperatures. Mineral elements were measured by Inductively Couple argon Plasma emission spectroscopy (Thermo Electron Corporation, Waltham, MA, USA).

### Secondary metabolite content and antioxidant activity of the wheat varieties

The extraction of soluble (free) and insoluble (bound) phenolic compounds was performed according to Dinelli *et al.*^7^ Total polyphenol content in both the free and bound fractions was measured using the spectrophotometric Folin–Ciocalteu method (Lambda 25 Spectrophotometer, Perkin Elmer Corporation, Waltham, MA, USA) with gallic acid as the reference standard.^14^ Similarly, the total flavonoid content was determined using a colorimetric method with catechin as the reference standard.^15^ Only the total polyphenol and flavonoid contents are, respectively, presented as the sum total of the free and bound fractions. Total carotenoid content was estimated from the yellow pigment content, extracted and measured according to a micro-method developed by Belaggi *et al.*^16^

Antioxidant activity in both the free and bound fractions was estimated using two different methods. The activities presented in the results similarly reflect the sum total of the free and bound fractions measured. The radical scavenging activity of antioxidant active substances in the extract was determined using the stable radical 2,2-diphenyl-1-picrylhydrazyl, which becomes reduced to 2,2-diphenyl-1-picrylhydrazyl, according to the spectrophotometric method of Brand-Williams *et al.*^17^ The efficient concentration (EC), representing the amount of antioxidant in the sample necessary to decrease the initial 2,2-diphenyl-1-picrylhydrazyl concentration by half (EC50), was calculated for each sample from a calibration curve by linear regression. The higher the EC50 the less efficient the antioxidant activity of the sample. For clarity, the EC50 value for each of the sample was converted into antiradical power, using the following equation antiradical power=1/EC50 × 100. The higher the antiradical power value the more efficient the radical scavenging activity of the sample.

The chelating activity on ferrous iron was estimated according to a modification of the spectrophotometric method of Köksal *et al.*^18^ Briefly, in 1 ml total, different volumes of sample were added to 100% (v/v) EtOH containing 75 μl 0.6 mℳ FeCl_2_. The reaction was initiated by the addition of 25 μl 5 mℳ ferrozine. The controls contained only ferrozine and FeCl_2_. The percentage inhibition of the ferrozine–Fe^2+^ complex was calculated using the following equation: ferrous ion-chelating effect (%)=(1−As/Ac) × 100 where Ac and As are the absorbance values of the controls and samples, respectively.

### Blood measurements

Venous blood samples were taken by the study physician after an overnight fasting, into evacuated plastic tubes (Vacutainer, BD, Oxford, UK). Samples, obtained by centrifuging at 3000 *g* for 15 min at 4 °C, were stored in aliquots at –80 °C until analysis. Lipid variables, blood glucose, liver enzymes and serum electrolytes were assessed by conventional methods. Pro- and anti-inflammatory cytokines were determined by using the Bio-Plex cytokine assay (Bio-Rad Laboratories Inc., Hercules, CA, USA), according to manufacturer's instructions.

### Thiobarbituric acid reactive substance assay and total antioxidant capacity

Plasma levels of malondialdehyde were quantified using the thiobarbituric acid reactive substance assay kit (Oxitek-ZeptoMetrix Corporation, Buffalo, NY, USA) following the manufacturer's protocol. Briefly, 100 μl of pure plasma was aliquoted in glass tubes (each sample was run in duplicate). Then, freshly prepared reaction buffers were added and tubes were placed in a heat block at 95 °C for 1 h. After incubation, samples were quickly cooled on ice and centrifuged at 3000 *g* for 15 min to remove debris. The fluorescence emission of the recovered supernatant was measured with an excitation wavelength of 530 nm and an emission wavelength of 550 nm, using a Perkin Elmer LS55 spectrofluorimeter. Thiobarbituric acid reactive substances were expressed in terms of malondialdehyde equivalent (nmol/ml). Total antioxidant capacity, accounting for total hydrophilic reactive oxygen species scavengers, was measured in plasma by a chemiluminescence assay using the photoprotein Pholasin (Abel Antioxidant Test Kit, Knight Scientific Ltd, Plymouth, UK). The protein content of samples was measured by using the Bradford assay.^19^ The results were calculated using an ℒ-ascorbic acid-based standard curve. Oxidative modification on plasma proteins was assessed on the basis of carbonyl content using two to four dinitrophenylhydrazine, as described by Levine *et al.*^20^

### Statistical analysis

Statistical analysis was performed by using the statistical package PASW 18.0 for Macintosh (SPSS Inc., Chicago, IL, USA). All variables were checked for normal distribution before data analysis. Data were expressed as arithmetic means±s.d. for normally distributed variables and as median and range or non-normally distributed data. The Mann–Whitney test was used for testing differences between the groups. One-way analysis of variance was used for testing differences between Kamut and control flour and semolina samples. Non-normally distributed data were log-transformed, and further analysis was carried out with the transformed data. The two interventions were analysed by taking into account both phases in the two groups of subjects at different stages. Carryover effect, that is, the effect that considers whether the impact of the first treatment is still present when the patient enters the second treatment period, were analysed with two models. First, we evaluated the period effect by comparing the geometric mean (and 95% confidence interval) of the difference between the evaluation at baseline and the evaluation at the end of the washout period, with the use of a paired *t*-test. Second, we evaluated the sequence effect, which considers whether the impact of Kamut treatment was different when the order of administration changed. This effect was estimated by comparing the geometric mean change difference between treatments in the Kamut group and in the control group, after adjustment for order of treatment. A general linear model for repeated measurements was performed to compare the effect of the two different treatments. *Post hoc* Bonferroni correction was applied to account for multiple comparisons. A model with adjustments for age, gender, smoking habit, hypertension and physical activity was performed. Data for the general linear model were reported as geometric mean and s.e. A *P*-value<0.05 was considered to indicate statistical significance.

## Results

### Characteristics of the wheat varieties

Flour and semolina were characterised for primary component composition, including fibre, protein, total and resistant starch, as well as for various secondary antioxidant metabolites and potential antioxidant activity. The major differences reported in the above-mentioned constituents were between the Kamut and control flour, or the part of the diet involving the consumption of bread, biscuits and crackers (Table 1). A significantly higher amylose/amylopectin ratio, protein content, antioxidant activity (polyphenols, flavonoids and carotenoids) as well as 2,2-diphenyl-1-picrylhydrazyl antiradical activity were apparent in the Kamut flour with respect to the control flour. With the exception of the amylose/amylopectin ratio in the Kamut flour, no differences in primary constituents, secondary metabolites or antioxidant activities were evident between Kamut flour and either the Kamut semolina or control semolina.

Flour and semolina were also characterised for various mineral elements. Both Kamut semolina and flour contained significantly higher content of minerals such as potassium, magnesium, phosphorus, zinc, iron and vanadium with respect to control semolina and flour (Table 2).

### Characteristics of the study population

Three participants were current smokers and two were hypertensive under an optimal therapeutic control. At the end of the intervention programme, blood pressure and BMI did not change significantly with respect to baseline in both groups (data not shown). No significant differences for demographic, clinical and laboratory parameters at baseline, between subjects randomized to consume either Kamut or control wheat products as the first intervention, were reported (data not shown).

### Modifications in lipid and metabolic profiles

**Figure 1:**
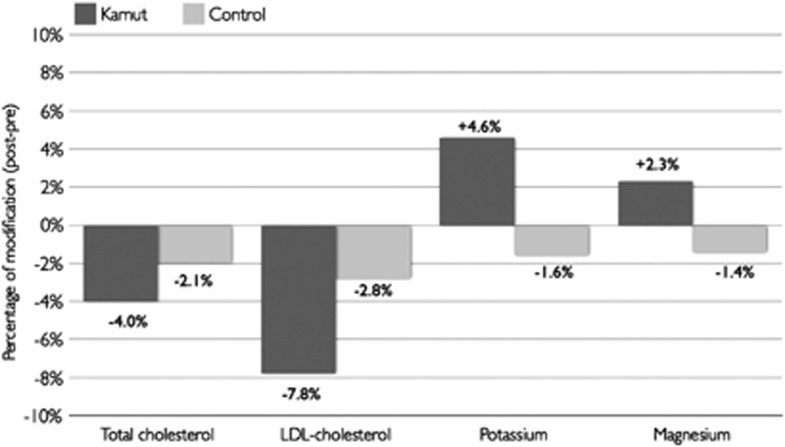
Mean percentage of change for selected metabolic parameters and minerals.

To test the possible effects of a replacement diet with Kamut products on the parameters investigated, a general linear model for repeated measurements, adjusted for age, gender, smoking habit, hypertension and physical activity was performed. In Table 3, adjusted values for BMI and all the variables investigated before and after the two dietary interventions were reported. During the phase of dietary replacement with Kamut products, participants experienced a significant amelioration of some parameters such as blood glucose, alanine aminotransferase, total cholesterol and low-density lipoprotein cholesterol. Indeed, total cholesterol decreased significantly by 4%, with a mean reduction of 8.46 mg/dl and low-density lipoprotein cholesterol by 7.8% with a mean reduction of 9.82 mg/dl, respectively. In contrast, no significant changes during the phase of dietary replacement with control wheat products were reported (Figure 1).

With regard to serum electrolytes, a significant increase of both potassium and magnesium levels was evident after the Kamut phase of dietary intervention, whereas no changes during the phase of intervention with the control wheat products were reported (Table 3 and Figure 1).

### Modifications in redox status

Of interest, two important parameters of oxidative stress, namely thiobarbituric acid reactive substances and carbonyls decreased significantly after the intervention phase of Kamut (Table 4). This effect was not evident after the consumption of control products.

### Modifications in inflammatory profile

**Figure 2:**
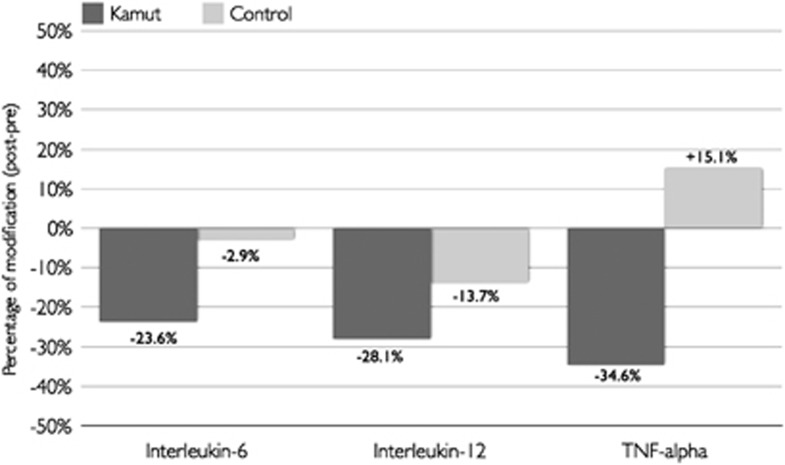
Mean percentage of change for selected inflammatory cytokines.

After the period of dietary replacement with Kamut products, a significant reduction in the circulating levels of some pro-inflammatory cytokines such as interleukin (IL)-6 (−23.6%), IL-12 (−28.1%), monocyte chemotactic protein-1 (−17%), macrophage inflammatory protein-1β (−14.7%), tumour necrosis factor-α (−34.6%) and vascular endothelial growth factor (−10.5%) was observed (Table 5). In contrast, after the period of intervention with the control products, the only significant reductions reported were for monocyte chemotactic protein-1 (−16.9%) and macrophage inflammatory protein-1β (−18.7%) levels (Figure 2).

Period and sequence carryover effects were not present for all the parameters investigated (data not shown).

## Discussion

Over the last years, widespread publicity promoting Kamut as a healthy grain alternative has necessitated the implementation of human trials to verify such claims. This study is the first human trial currently being performed to test the possible efficacy of Kamut products on cardiovascular biomarkers. Various biochemical, lipid, antioxidant and inflammatory parameters related to cardiovascular disease risk in adult humans were investigated following the adoption of a diet of organic, semi-whole-wheat Kamut products. Results hypothesise that after ingesting products made from Kamut, improvements for some blood parameters such as minerals (potassium, magnesium and glucose), metabolic biomarkers (alanine aminotransferase, total cholesterol and low-density lipoprotein cholesterol), parameters of redox status (lipid peroxidation and oxidised carbonyl proteins) as well as inflammatory cytokines (IL-6, IL-12, tumour necrosis factor-α and vascular endothelial growth factor) could be obtained.

Of the principle results of our study were the reductions observed for total and low-density lipoprotein cholesterol levels after intervention period with Kamut products. In this study, no differences in the dietary fibre and resistant starch content between Kamut and control semolina/flour were evident. Hence, dietary fibre and resistant starch alone were clearly not instrumental in improving these metabolic parameters. Similarly, no significant differences for triglycerides were observed. Actually, high levels of triglycerides are important risk factors for cardiovascular disease not only for the metabolic and biochemical properties but also because they are a mirror of the incorrect dietary habits. In our study population, only small nonsignificant changes for triglycerides' levels were obtained, allowing us to hypothesise that subjects did not modify their diets during the study.

Interestingly, in our investigation, we found that Kamut contains an elevated mineral content and that potassium and magnesium contents in the Kamut semolina/flour were significantly higher than that of the control. This difference was positively associated with an increase in blood potassium and magnesium after the ingestion of Kamut brand products.

Notably, in this study significant improvements on various aspects of the inflammatory profile were observed after ingesting the Kamut products. In particular, monocyte chemotactic protein-1 and macrophage inflammatory protein-1β were significantly reduced both after consumption of Kamut and the control. Actually, monocyte chemotactic protein-1 expression is essential for the earliest inflammatory response, which is marked by the recruitment of monocytes into the subendothelium.^21^ Moreover, IL-8, an additional chemokine and early marker of inflammation, was unchanged after consumption of both Kamut and the control showing that diet of certain foods may have a role in the regulation of different types of cytokine molecules. The significant reduction in IL-6, tumour necrosis factor-α and vascular endothelial growth factor only after consumption of Kamut indicates that the effect of the latter was potentially more rapid in improving the inflammatory response downstream of the earliest markers. In particular, IL-6 and tumour necrosis factor-α are the key pro-atherogenic cytokines, which induce the expression of a vast array of inflammatory responses of endothelial cells and macrophages, whereas vascular endothelial growth factor enhances atherosclerotic plaque formation.^22, 23, 24^

Furthermore, after the dietary intervention phase with Kamut, protection against oxidative stress was observed in the blood, although the carotenoid and polyphenol (flavonoid) content and antiradical activity were equivalent in the Kamut semolina/flour and control semolina (used for pasta) with only significantly lower levels in the control flour. However, not all antioxidant substances were measured in this study, and it is likely that additional compounds in Kamut may have improved protection against oxidative stress in blood. Similarly, these antioxidant compounds are also instrumental for anti-inflammatory properties.^25^

Although the results are promising, the number of participants (22 in total) represents a limitation of this study. Further and larger studies need to be conducted before drawing any firm conclusion on the effects of such food products on human health. We are aware that changes in dietary and/or lifestyle habits could have affected parameters investigated. However, before initiating, all subjects were instructed by physicians and by an expert dietician to maintain their usual lifestyle habits.

In conclusion, the preliminary results of this study hypothesise that Kamut could afford health benefits by improving metabolic, lipid, antioxidant and inflammatory blood profiles. For the first time, the findings of this study provide data suggesting the benefits of Kamut. These results promote research to fully elucidate the link between specific components of the wheat and their beneficial effects in reducing several cardiovascular risk markers. However, results must be interpreted with caution because significant differences obtained on the experimental arm but not on the control arm do not necessarily mean that treatment has had a real effect. Further studies are needed to draw conclusive findings.

## Figures and Tables

**Table 1:** Composition of Kamut and control wheat

*Variable*	*Kamut (Semolina)*	*Control (Semolina)*	P-value	*Kamut (Flour)*	*Control (Flour)*	P-value
Protein, %	16.07±0.05	16.89±0.15	0.1	16.36±0.03	13.98±0.38	0.05
Total fibre, %	9.78±1.21	9.99±1.08	0.1	8.96±1.62	9.64±0.24	0.1
Total starch, %	64.74±3.12	66.82±2.54	0.7	71.34±1.71	73.50±0.22	0.2
Amylose, %	27.16±0.48	27.51±0.79	0.7	34.50±3.69	28.12±0.89	0.05
Amylopectin, %	72.84±0.48	72.49±0.79	0.7	65.50±3.67	71.88±0.89	0.05
Amylose/amylopectin	0.372	0.397	0.7	0.530	0.391	0.04
DPPH	126.52±10.88	118.89±10.31	0.4	115.39±10.74	104.07±6.50	0.04
Fe^2+^ chelation, %	59.38±8.63	40.63±9.24	0.05	21.57±4.43	8.98±4.72	0.03
Polyphenols, mg/g DM	1.62±0.13	1.59±0.13	0.7	1.67±0.16	1.21±0.10	<0.00001
Carotenoids, mg/g DM	14.71±0.24	15.09±0.96	0.6	15.29±2.30	6.38±0.32	0.008
Flavonoids, mg/g DM	0.46±0.08	0.43±0.03	0.5	0.34±0.03	0.20±0.03	0.05

Abbreviation: DPPH, 2,2-diphenyl-1-picrylhydrazyl.

Data are reported as mean±s.d.

**Table 2:** Mineral element composition of Kamut and control wheat

*Variable (mg/kg)*	*Kamut (Semolina)*	*Control (Semolina)*	P-value	*Kamut (Flour)*	*Control (Flour)*	P-value
Potassium	2817±6.52	2393±0.808	0.006	2663±0.811	1553±6.47	0.001
Magnesium	909.57±58.7	795.58±50.1	0.003	889.03±27.6	542.06±28.9	0.001
Phosphorus	2.98±0.26	2.67±0.62	0.001	2.85±0.62	1.77±0.84	0.02
Zinc	25.19±0.05	25.99±0.09	0.02	24.95±0.02	15.15±0.05	0.001
Iron	29.63±0.24	28.02±0.04	0.06	24.13±0.04	20.42±0.14	0.01
Selenium	0.99±0.04	0.92±0.03	0.2	0.90±0.008	0.74±0.006	0.02
Vanadium, mg/kg	1.01±0.02	0.73±0.008	0.005	0.98±0.008	0.63±0.004	0.0001

Data are reported as mean±s.d.

**Table 3:** Modifications of biochemical parameters

*Variable*	*Kamut Pre*	*Kamut post*	*Change*	P-value	*Control pre*	*Control post*	*Change*	P-value
White blood cells, *n*	6.09 (5.51–6.68)	6.05 (5.43–6.66)	−0.04 (−0.42; 0.33)	0.8	6.25 (5.54–6.97)	6.45 (5.64–7.26)	0.19 (−0.40; 0.80)	0.5
Fibrinogen, mg/dl	391.3 (362.9–420.4)	403.4 (371.8–435)	12.1 (−11.9; 36.2)	0.3	396.2 (368.4–423.9)	388.1 (361.9–414.3)	−8.15 (−24.1; 7.82)	0.3
Blood glucose, mg/dl	81.1 (77.3–84.9)	78.1 (75.5–80.7)	−3.00 (−5.78; −0.23)	0.04	80.3 (76.8–83.7)	79.3 (76.4–82.3)	−0.90 (−4.16; 2.33)	0.6
Insulin, mU/l	6.99 (5.33–8.66)	9.29 (6.11–12.46)	2.29 (−0.66; 5.25)	0.1	9.84 (6.49–13.18)	9.82 (6.03–13.60)	−0.02 (−2.81; 2.76)	0.9
AST, U/l	21.4 (19.9–22.8)	20.8 (18.9–22.7)	−0.55 (−2.37; 1.28)	0.5	22.8 (20.1–25.5)	24.7 (21.6–27.7)	1.91 (−0.93; 4.74)	0.2
ALT, U/l	21.6 (19.4–23.9)	19.4 (17.1–21.6)	−2.32 (−4.32; −0.31)	0.03	21.1 (18.2–24.1)	23.1 (20.3–25.8)	1.90 (−0.38; 4.18)	0.09
Total cholesterol, mg/dl	210.4 (192.8–227.9)	201.9 (185.4–218.4)	−8.46 (−15.96; −0.95)	0.03	207.1 (193.5–220.6)	202.7 (187.9–217.5)	−4.38 (−11.64; 2.88)	0.2
LDL cholesterol, mg/dl	125.5 (110.3–140.7)	115.6 (103.7–127.6)	−9.82 (−18.23; −1.41)	0.02	122 (108.7–135.4)	118.6 (102.2–134.9)	−3.42 (−7.69; 0.85)	0.1
HDL-cholesterol, mg/dl	60.2 (53.2–67.3)	58.2 (50.8–65.7)	−2.00 (−4.73; 0.73)	0.1	58.2 (49.4–66.9)	60.8 (52.2–69.4)	2.62 (−1.61; 6.85)	0.2
Triglycerides, mg/dl	124.5 (93.1–155.9)	134.2 (95.2–173.2)	9.64 (−15.63; 34.90)	0.4	135.1 (99.1–171.1)	122.7 (87.7–157.7)	−12.42 (−55.95; 31.10)	0.5
Sodium, mEq/l	140.1 (139.3–140.9)	140.4 (139.6–141.1)	−0.23 (−0.52; 0.97)	0.5	139.9 (139.2–140.6)	140.2 (139.4–141)	0.29 (−0.46; 1.04)	0.4
Potassium, mEq/l	4.17 (4.03–4.32)	4.37 (4.25–4.49)	0.20 (0.07; 0.32)	0.005	4.25 (4.09–4.42)	4.18 (4.03–4.33)	−0.07 (−0.31; 0.17)	0.5
Magnesium, mg/dl	2.16 (2.09–2.22)	2.21 (2.13–2.29)	0.05 (0.002; 0.09)	0.04	2.14 (2.07–2.21)	2.11 (2.04–2.17)	−0.03 (−0.10; 0.04)	0.3
Iron, μg/dl	112.6 (91.2–133.9)	88.6 (73.4–103.7)	−24.1 (−49.4; 1.35)	0.06	92.5 (77.2–107.8)	83.1 (68.2–98.1)	−9.33 (−27.76; 9.09)	0.3

Abbreviations: ALT, alanine aminotransferase; AST, aspartate aminotransferase; HDL, high-density lipoprotein; LDL, low-density lipoprotein.

Data are reported as geometric mean and (range). General linear model adjusted for age, gender, smoking habit, hypertension and physical activity.

**Table 4:** Modifications of redox status

*Variable*	*Kamut Pre*	*Kamut post*	*Change*	P-value	*Control pre*	*Control post*	*Change*	P-value
TBARs, nmol/ml	0.79 (0.63–0.95)	0.62 (0.52–0.72)	−0.17 (−0.34; −0.09)	0.04	0.66 (0.55–0.75)	0.75 (0.57–0.94)	0.09 (−0.09; 0.29)	0.3
TAC, nmol/ml	317.9 (278–357.7)	327.2 (281.6–372.8)	9.31 (−15.27; 33.90)	0.4	345.4 (307.9–382.9)	300.6 (260.9–340.4)	−44.75 (−73.89; −15.61)	0.005
Carbonyls, nmol/ml	0.91 (0.74–1.03)	0.75 (0.63–0.87)	−0.16 (−0.31; −0.01)	0.03	0.78 (0.62–0.95)	0.89 (0.64–1.06)	0.11 (−0.07; 0.30)	0.2

Abbreviations: TAC, total antioxidant capacity; TBARs, thiobarbituric acid reactive substances.

Data are reported as geometric mean and (range). General linear model adjusted for age, gender, smoking habit, hypertension and physical activity.

**Table 5:** Modifications of inflammatory profile

*Variable (pg/ml)*	*Kamut Pre*	*Kamut post*	*Change*	P-value	*Control pre*	*Control post*	*Change*	P-value
IL-1ra	36.1 (0.78–71.4)	22.9 (3.48–42.5)	−13.06 (−29.1; 3.00)	0.1	24.6 (11.1–38.1)	17.4 (11.3–23.3)	−7.26 (−16.3; 1.81)	0.1
IL-4	0.34 (0.20–0.48)	0.29 (0.16–0.43)	−0.05 (−0.09; 0.005)	0.07	0.37 (0.23–0.51)	0.31 (0.19–0.43)	−0.06 (−0.17; 0.04)	0.2
IL-6	3.56 (2.73–4.39)	2.72 (2.23–3.21)	−0.84 (−1.52; −0.16)	0.02	3.13 (2.28–3.98)	3.05 (2.24–3.86)	−0.09 (−0.74; 0.57)	0.9
IL-8	32.04 (13.5–50.6)	22.8 (11.6–47.4)	−9.18 (−28.7; 10.4)	0.3	23.2 (10.8–35.6)	22.1 (6.7–37.6)	−1.05 (−8.01; 5.90)	0.8
IL-10	7.30 (3.13–11.5)	4.76 (2.92–6.59)	−2.55 (−5.57; 0.47)	0.09	4.82 (3.90–5.74)	4.34 (3.29–9.38)	−0.48 (−1.50; 0.54)	0.3
IL-12	11.7 (8.99–14.4)	8.39 (6.37–10.4)	−3.29 (−4.81; −1.78)	<0.0001	10.7 (9.19–12.28)	9.27 (7.64–10.89)	−1.47 (−3.26; 0.33)	0.1
IL-17	3.11 (2.35–3.87)	2.61 (1.85–3.38)	−0.49 (−1.29; 0.31)	0.2	3.17 (2.15–4.19)	2.54 (1.39–3.70)	−0.63 (−1.53; 0.27)	0.2
IP-10	837.2 (586.3–1088)	807.1 (579.7–1034.6)	−30 (−95.9; 35.9)	0.3	863.1 (562.9–1163.4)	838.3 (610.3–1066.2)	−24.9 (−225.9; 176.2)	0.8
MCP-1	54 (38.2–69.8)	44.8 (32.7–56.9)	−9.20 (−16.9; −1.42)	0.02	55.4 (38.9–71.8)	45.9 (31.6–60.4)	−9.37 (−15.4; −3.34)	0.005
MIP-1β	171.3 (114.7–200.8)	146.2 (117.7–174.7)	−25.1 (−42.6; −7.55)	0.008	169.8 (133.1–206.5)	138.1 (113.4–162.9)	−31.7 (−48.8; −14.6)	0.001
TNF-α,	8.27 (5.18–11.4)	5.42 (3.82–7.01)	−2.86 (−5.63; −0.09)	0.04	6.34 (4.22–8.45)	7.47 (4.39–10.5)	1.13 (−2.56; 4.82)	0.5
VEGF	77.5 (64.9–90.2)	69.3 (54.2–84.5)	−8.18 (−16.0; −3.43)	0.04	84.4 (71.3–97.5)	74.9 (58.3–91.4)	−9.54 (−20.5; 1.40)	0.08

Abbreviations: IP-10, interferon gamma-induced protein-10; MCP-1, monocyte chemotactic protein-1; MIP-1β, macrophage inflammatory protein-1β TNF-α, tumour necrosis factor-α VEGF, vascular endothelial growth factor. Data are reported as geometric mean and (range). General linear model adjusted for age, gender, smoking habit, hypertension and physical activity.

## References

[bib1] Hasler CM Brown AC American Dietetic Association Position of the American Dietetic Association: functional foods J Am Diet Assoc 2009 109 735 746 19338113 10.1016/j.jada.2009.02.023

[bib2] Sofi F Abbate R Gensini GF Casini A Accruing evidence about benefits of adherence to Mediterranean diet on health: an updated systematic review with meta-analysis Am J Clin Nutr 2010 92 1189 1196 20810976 10.3945/ajcn.2010.29673

[bib3] Fardet A New hypotheses for the health protective mechanisms of whole-grain cereals: what is beyond the fibre Nutr Res Rev 2010 23 65 134 20565994 10.1017/S0954422410000041

[bib4] Shewry PR Wheat J Exp Bot 2009 60 1537 1553 19386614 10.1093/jxb/erp058

[bib5] Gil A Ortega RM Maldonado J Whole grain cereals and bread: a duet of the Mediterranean diet for the prevention of chronic diseases Public Health Nutr 2011 14 2316 2322 22166190 10.1017/S1368980011002576

[bib6] Truswell AS Cereal grains and coronary heart disease Eur J Clin Nutr 2002 56 1 14 11840174 10.1038/sj.ejcn.1601283

[bib7] Sofi F Ghiselli L Cesari F Gori AM Mannini L Casini A Effects of short-term consumption of bread obtained by an old Italian grain variety on lipid, inflammatory, and haemorheological variables: an intervention study J Med Food 2010 13 1 6 20438321 10.1089/jmf.2009.0092

[bib8] Marotti I Bregola V Aloisio I Di Gioia D Bosi S Di Silvestro R Prebiotic effect of soluble fibers from modern and old durum-type wheat varieties on *Lactobacillus* and *Bifidobcaterium* strains J Sci Food Agric 2012 92 2133 2140 22298124 10.1002/jsfa.5597

[bib9] Benedetti S Primiterra M Tagliamonte MC Carnevali A Gianotti A Bordoni A Counteraction of oxidative damage in the rat liver by an ancient grain (Kamut brand khorasan wheat) Nutrition 2012 28 436 441 22129853 10.1016/j.nut.2011.08.006

[bib10] Prosky L Asp N-G Schweizer TF De Vries JW Furda L Determination of insoluble, soluble, and total dietary fibre in foods and food products J Assoc Off Anal Chem 1988 71 1017 1023 2853153

[bib11] Lee SC Prosky L De Vries JW Determination of total, soluble and insoluble fibre in foods-enzymatic–gravimetric method J Assoc Off Anal Chem 1992 75 395 416

[bib12] McClerry BV Monaghan DA Measurement of resistant starch J AOAC Int 2002 85 665 675 12083259

[bib13] Yun SH Matheson NK Estimation of amylase content of starches after precipitation of amylopectin by Concanavalin A Starch 1990 42 302 305

[bib14] Singleton VL Orthofer R Lamuela-Raventos RM Analysis of total phenols and other oxidation substrates and antioxidants by means of Folin–Ciocalteu reagent Methods Enzymol 1999 299 152 178

[bib15] Adom KK Sorrells ME Liu RH Phytochemical profiles and antioxidant activity of wheat varieties J Agric Food Chem 2003 51 7825 7834 14664553 10.1021/jf030404l

[bib16] Belaggi R Platani C Nigro F Cattivelli L A micro-method for the determination of yellow pigment content in durum wheat J Cereal Sci 2010 52 106 110

[bib17] Brand-Williams W Cuvelier ME Berset C Use of a free radical method to evaluate antioxidant activity Lebensm Wiss U Technol 1995 28 25 30

[bib18] Köksal E Gülçin I Beyza S Sarikaya Ö Bursal E *In vitro* antioxidant activity of silymarin J Enzyme Inhib Med Chem 2009 24 395 405 18830883 10.1080/14756360802188081

[bib19] Bradford MM A rapid and sensitive method for the quantitation of microgram quantities of protein utilizing the principle of protein-dye binding Anal Biochem 1976 72 248 254 942051 10.1016/0003-2697(76)90527-3

[bib20] Levine RL Williams JA Stadtman ER Shacter E Carbonyl assays for determination of oxidatively modified proteins Methods Enzymol 1994 233 346 357 8015469 10.1016/s0076-6879(94)33040-9

[bib21] Shin WS Szuba A Rockson SG The role of chemokines in human cardiovascular pathology: enhanced biological insights Atherosclerosis 2002 160 91 102 11755926 10.1016/s0021-9150(01)00571-8

[bib22] Kofler S Nicekel T Weis M Role of cytokines in cardiovascular diseases. A focus on endothelial responses to inflammation Clinical Sci 2005 108 205 213 15540988 10.1042/CS20040174

[bib23] Tousoulis D Antonaides C Stefanadis C Assessing inflammatory status in cardiovascular disease Heart 2007 93 1001 1007 17639118 10.1136/hrt.2006.088211 PMC1994393

[bib24] Esposito K Giugliano D Diet and inflammation: a link to metabolic and cardiovascular disease Eur Heart J 2006 27 15 20 16219650 10.1093/eurheartj/ehi605

[bib25] Santangelo C Varì R Scazzocchio B Di Benedetto R Filesi C Masella R Polyphenols intracellular signalling and inflammation Ann Ist Sup Sanita 2007 43 394 405 18209273

